# Patient reported impact of symptoms in amyotrophic lateral sclerosis (PRISM-ALS): A national, cross-sectional study

**DOI:** 10.1016/j.eclinm.2022.101768

**Published:** 2022-12-13

**Authors:** Christine Zizzi, Jamison Seabury, Spencer Rosero, Danae Alexandrou, Ellen Wagner, Jennifer S. Weinstein, Anika Varma, Nuran Dilek, John Heatwole, Joanne Wuu, James Caress, Richard Bedlack, Volkan Granit, Jeffrey M. Statland, Paul Mehta, Michael Benatar, Chad Heatwole

**Affiliations:** a Center for Health + Technology, 265 Crittenden Blvd, CU 420694, Rochester, NY, 14642, USA; b University of Rochester, Department of Neurology, 601 Elmwood Ave, Box 673, Rochester, NY, 14642, USA; c Sutherland High School, Pittsford, NY, USA; d University of Miami Miller School of Medicine, Department of Neurology, 1120 NW 14th Street, Suite 1300, Miami, FL, 33136, USA; e Wake Forest Baptist Health, Medical Center Blvd, Winston–Salem, NC, 27157, USA; f Duke University School of Medicine, Department of Neurology, 311 Research Dr, Durham, NC, 27710, USA; g University of Kansas Medical Center, Department of Neurology, 3901 Rainbow Blvd, Kansas City, KS, 66160, USA; h Centers for Disease Control and Prevention/Agency for Toxic Substances and Disease Registry, National ALS Registry, 4770 Buford Highway NE, Atlanta, GA, 30341, USA

**Keywords:** Amyotrophic lateral sclerosis, Patient-reported outcomes, PRISM, Disease burden, Motor neuron disease

## Abstract

**Background:**

As novel therapeutic interventions are being developed and tested in the amyotrophic lateral sclerosis (ALS) population, there is a need to better understand the symptoms and issues that have the greatest impact on the lives of individuals with ALS. We aimed to determine the frequency and relative importance of symptoms experienced by adults in a national ALS sample and to identify factors that are associated with the greatest disease burden in this population.

**Methods:**

We conducted 15 qualitative interviews of individuals with varied ALS phenotypes and analyzed 732 quotes regarding the symptomatic disease burden of ALS between August 2018 and March 2019. We subsequently conducted a national, cross-sectional study of 497 participants with ALS and ALS variants through the Centers for Disease Control and Prevention's (CDC) National ALS Registry between July 2019 and December 2019. Participants reported on the prevalence and relative importance of 189 symptomatic questions representing 17 symptomatic themes that were previously identified through qualitative interviews. Analysis was performed to determine how age, sex, education, employment, time since onset of symptoms, location of symptom onset, feeding tube status, breathing status and speech status relate to symptom and symptomatic theme prevalence.

**Findings:**

Symptomatic themes with the highest prevalence in our sample were an inability to do activities (93.8%), fatigue (92.6%), problems with hands or fingers (87.7%), limitations with mobility or walking (86.7%), and a decreased performance in social situations (85.7%). Participants identified inability to do activities and limitations with mobility or walking as having the greatest overall effect on their lives.

**Interpretation:**

Individuals with ALS experience a variety of symptoms that affect their lives. The prevalence and importance of these symptoms differ among the ALS population. The most prevalent and important symptoms offer potential targets for improvements in future therapeutic interventions.

**Funding:**

Research funding was provided by 10.13039/100000971ALS Association.

**Research in context**

**Evidence before this study**

Amyotrophic lateral sclerosis (ALS) is the most common adult motor neuron disease. Prior studies have attempted to explore patient-reported disease burden in this population; however, these studies have largely been limited in magnitude and geographical distribution. We searched PubMed for articles from inception to May 2022, using a combination of the following search terms: “amyotrophic lateral sclerosis,” “patient-reported,” “disease burden,” “symptoms.” We identified one article aimed at determining which symptoms were most problematic for patients with ALS and how the severity changed over time. This retrospective study analyzed data from a randomized, double-blind, placebo-controlled trial of ceftriaxone in ALS with 82 participants. A second study with 65 participants with sporadic ALS from Southwestern China evaluated pain from perspectives of ALS patients through three questionnaires: numerical pain rating scale (NRS), Brief Pain Inventory (BPI), and Douleur Neuropathique-4 (DN4). Still another study has utilized 40 participants to evaluate the association between three patient-reported outcome measures (PROMs) in ALS—PROMIS-10, NeuroQol-fatigue, and ALSFRS-R. Collectively, prior research has identified and highlighted that there is a high level of disease burden in ALS. However, the quantification and comparison of all of the most important symptoms that occur in this condition has been limited.

**Added value of this study**

To our knowledge, this is the largest study to systematically assess the multifactorial patient-reported disease burden in ALS while simultaneously evaluating the effects of demographic features on ALS symptomatic themes through a partnership with the Centers for Disease Control and Prevention's (CDC) National ALS Registry.

**Implications of all the available evidence**

Results from our large, national cross-sectional study further defines the multifactorial clinical phenotype of patients with ALS using extensive patient input. This research and the data it has generated has implications for future therapeutic trials and the clinical care of patients with ALS. The ALS clinical and research communities can now review the relative importance and prevalence of numerous symptoms in ALS and utilize this data to identify undertreated areas of disease burden and potential targets for future therapeutic interventions in this population. In addition, this data provides an insight into which subpopulations experience the greatest disease burden and how disease burden changes based on patients’ demographic information and functional performance.

## Introduction

Amyotrophic lateral sclerosis (ALS) is a progressive, heterogeneous neurodegenerative disorder characterized by the degeneration of both upper and lower motor neurons, as well as associated frontotemporal spectrum dysfunction.^1^^,^^2^ Patients experience progressive muscular atrophy and weakness, typically leading to respiratory failure and death within three to five years.^3^ ALS is the most common motor neuron disease in adults. In the United States, the incidence is approximately 1–2.6 cases per 100,000 people annually.^4^ As of 2016, ALS was estimated to have an age-adjusted prevalence rate of 5.2 per 100,000 people.^5^

In preparation for future clinical trials and other therapeutic development efforts, it is important to have a clear understanding of the symptoms and issues that have the greatest effect on individuals with ALS. Changes in the symptoms that matter most to individuals with ALS may serve as a benchmark by which to judge the efficacy of future experimental therapeutics. Additionally, in the clinical setting, it is important to have a clear understanding of what symptoms are most common and important from the ALS patient's point of view in order to facilitate the management and care of patients.

Here, we use patient interviews and partner with the Centers for Disease Control and Prevention's (CDC) National ALS Registry to conduct a large, national cross-sectional survey to determine the prevalence and relative importance of symptoms and symptomatic themes in the ALS population.

## Methods

### Study participants

Eligible participants in this study included those aged 18 years or older with a diagnosis of motor neuron disease. Participants in phase 1 were those who had participated in the Phenotype Genotype Biomarker Study (NCT02327845) of the Clinical Research in ALS and related disorders for Therapeutic Development (CReATe) Consortium or were patients cared for at the University of Rochester ALS subspecialty clinic. All participants in phase 2 were registered and consenting members of the CDC's National ALS Registry.

### Study design

#### Phase 1: semi-structured qualitative interviews

We conducted interviews with participants with ALS by phone or in person. Using open-ended questions, we asked participants to identify the symptoms of ALS that have the greatest impact on their lives (Supplemental Interview Guide). All but one interviews were audio-recorded, transcribed, coded, and analyzed with a qualitative framework technique, triangulation, and an investigator consensus approach. One in-person interview was conducted with a non-verbal ALS participant who wrote responses on a white-board.

**Fig. 1:**
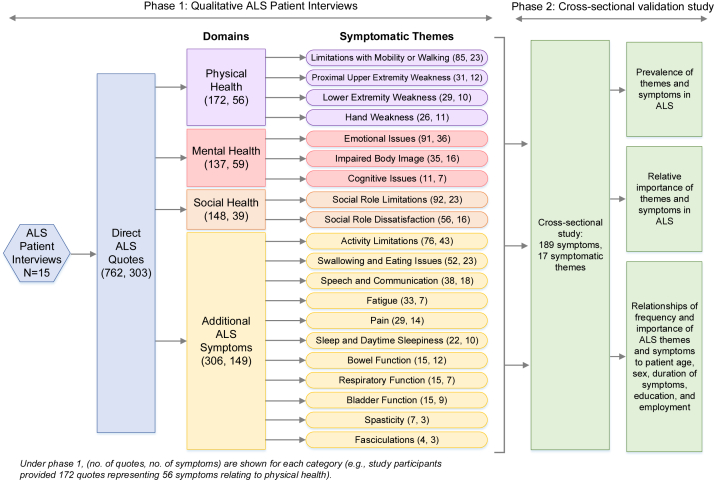
Overview of study design.

Reoccurring similar quotes among the interviewees were used to identify potentially relevant symptoms. Symptoms were then categorized into symptomatic themes, concepts representing a group of common symptoms. Each theme was categorized into a broader category of physical, mental, social or ALS disease-specific components of health, in accordance with the World Health Organization's framework of health (Fig. 1). All interviews were conducted between August 2018 and March 2019.

#### Phase 2: national cross-sectional study of motor neuron disease patients

We conducted an online cross-sectional study of those with ALS and ALS variants to identify those symptoms and symptomatic themes with the greatest importance to this population. Participants were recruited through the CDC's National ALS Registry. This registry requires members to have a diagnosis of ALS. Members of the registry who met our inclusion criteria were emailed a recruitment letter and a link to our survey on REDCap, a Health Insurance Portability and Accountability Act-compliant electronic data capture system. After clicking the link, potential participants were asked to read an information letter prior to beginning the survey. Participants were given the opportunity to complete the survey online, on paper, or over the phone. Participants then proceeded to a brief demographics questionnaire prior to accessing the main survey. Participants were asked not to complete the survey more than once. This was a survey for individuals with ALS and ALS variants, not caregivers.

Our cross-sectional survey included questions based on the symptoms and symptomatic themes previously identified by individuals with ALS in phase 1 as having a potentially high level of importance. Additional questions pertaining to potential symptoms of importance identified in other neurologic populations were included in the survey.^6^, ^7^, ^8^, ^9^, ^10^, ^11^ Question selection was determined with a consensus approach involving our research team, investigator members of the CReATe Consortium, and the CDC. In total, the survey sought information regarding 189 symptomatic questions representing 17 symptomatic themes. For each symptomatic question, the survey inquired, “How much does the following impact your life now?” Participants were provided with a 6-point Likert-type scale ranging from one to six. Likert scale options included the following: (1) I do not experience this; (2) I experience this but it does not affect my life; (3) It affects my life a little; (4) It affects my life moderately; (5) It affects my life very much; and, (6) It affects my life severely. Participants were given the option not to answer any question. At the end of the survey, participants were asked to list and rate the severity of any symptoms of importance not otherwise included in the survey. Participant data were included in the analysis from those who responded to at least one demographic question and one symptom question.

Our REDCap survey was active from July 2019 to December 2019. This methodology has previously been used and described in studies of other neurologic disease populations.^6^, ^7^, ^8^, ^9^, ^10^, ^11^

### Statistical analysis

We determined the prevalence of each symptom and symptomatic theme in our sample. We determined the average life impact (the relative importance) scores by calculating the mean of all scores of participants who experienced the symptom or symptomatic theme. The average life impact metric had a range of zero to four and was determined by assigning a numerical value to each participant response, as follows: I experience this but it does not affect my life = 0; It affects my life a little = 1; It affects my life moderately = 2; It affects my life very much = 3; It affects my life severely = 4. Additionally, a population impact score was calculated for each symptom and symptomatic theme by multiplying the percentage of participants with the symptom or theme (prevalence) by its average life impact score. The population impact score had a range of 0–4, with a value of 4 representing a symptom that affects all individuals with ALS at the highest level.

Responses were categorized based on the following demographic categories: (1) Sex (male, female); (2) Age (<63.3 years, >63.3 years); (3) Education level (4-year college and above, less than 4-year college); (4) Employment status (employed, unable to work); (5) Time since first signs of weakness ( ≤ 34 months, >34 months); (6) Location of symptom onset (limb onset, bulbar onset); (7) Speech status (able to talk clearly with no changes in speech, unable to talk clearly without changes in speech); (8) Feeding tube use (yes, no); and, (9) Breathing status (breathe without assistive ventilation, breathe with assistive ventilation). Group categorization was predetermined for each non-numerical category (sex, education level, employment status, location of symptom onset, speech status, feeding tube status, and breathing status). Numerical categories were split at the mean value (age, time since first signs of weakness). Additionally, we performed subgroup analysis between type of ALS: ALS, progressive muscular atrophy (PMA), primary lateral sclerosis (PLS), and ALS with frontotemporal dementia.

We obtained descriptive statistics for the prevalence and life impact of each symptomatic theme for the entire sample and for each subgroup. We then used Fisher exact tests to compare the prevalence of each theme across the different subgroup. In order to correct for multiple comparisons, the Benjamini-Hochberg procedure was used with a false discovery rate of 0.05 and 204 test statistics. As outlined by this method, the 204 p values are sorted from smallest to largest and the largest value of i such that p(i) ≤ 0.05 i/204 is determined. The null hypotheses associated with the p values p(1), …, p(i) are rejected, resulting in i discoveries.

### Ethics statement

All study activities were approved by the University of Rochester Institutional Review Board (STUDY0003775) and qualified for exemption due to research holding out no more than minimal risk to participants. As per the exempt status of this study, participants were not required to provide a written informed consent. As directed by the University of Rochester Institutional Review Board, all interview participants (phase 1 participants) and cross-sectional study participants (phase 2 participants) received and reviewed a detailed information letter prior to their involvement in this research. The detailed information letter disclosed the following information: description of research study, participation is voluntary, activities involved in the research, and the name and contact information of the study team and the IRB.

### Role of the funding source

The funders of the study had no role in study design, data collection, data analysis, data interpretation, writing of the report, or decision to publish. All authors had access to the data and approved the final manuscript for submission.

## Results

### Phase 1: semi-structured qualitative interviews

We conducted interviews with 14 participants with ALS by phone and 1 participant with ALS in person. Length of interviews were typically between 30 and 45 minutes. Through these qualitative interviews of 15 individuals with ALS, symptom saturation was met, few new concepts were mentioned, and we obtained 732 direct quotes. Participants identified 303 symptoms of potential importance to the larger ALS population. Of these identified symptoms, 189 symptomatic questions across 17 symptomatic themes were selected to be included in a survey sent to participants in phase 2 of the study (Fig. 1).

### Phase 2: national cross-sectional study of motor neuron disease participants

The CDC's National ALS Registry sent out recruitment email notifications on July 22, 2019 and October 24, 2019 to 11,373 and 11,836 participants, respectively. Four-hundred and ninety-seven participants from 44 U.S. states completed the demographics section and at least one question in the main survey. Demographic data for the sample are summarized in Table 1. Out of these 497 respondents, 447 respondents completed the last survey question. Supplemental Table S1 shows the number of responses for each survey question.

**Table 1:** Demographic information.

**No. of participants**	N	497
**Sex**		
Male	N (%)	309 (62.2)
Female		187 (37.6)
Other		1 (0.2)
**Age**	Mean (SD), Range	63.3 (9.6), 32 - 87
**Race**		
American Indian/Alaska Native	N (%)	0 (0)
Asian		4 (0.8)
Native Hawaiian/Pacific Islander		0 (0)
Black/African American		9 (1.8)
White		475 (95.6)
Other		7 (1.4)
Omitted		2 (0.4)
**Ethnicity, Hispanic/Latino**	N (%)	15 (3)
**Employment status**		
Employed for wages	N (%)	56 (11.3)
Self-employed		18 (3.6)
Out of work; not currently looking for work		4 (0.8)
Homemaker		1 (0.2)
Retired		254 (51.1)
Unable to work		164 (33.0)
**Level of education**		
Grade school	N (%)	2 (0.4)
High school		81 (16.3)
Technical degree		44 (8.9)
College		220 (44.3)
Master's or doctorate		150 (30.2)
**Months since diagnosis**	Mean (SD), Range	36.1 (47.1), 0 - 368
**Months since first noticing weakness**	Mean (SD), Range	51.6 (54.2), 3 - 384
**Location(s) of symptom onset (**participants could respond to more than one location)		
In the leg or lower extremity	N	230
In the arm or upper extremity		155
With swallowing (e.g. coughing, choking when swallowing)		28
With talking (e.g. slurred speech)		98
With breathing (e.g. shortness of breath)		17
Other		13
**Type of motor neuron disease**		
ALS	N (%)	434 (87.3)
ALS with Frontotemporal Dementia		10 (2.0)
Kennedy's disease (SBMA)		3 (0.6)
Primary lateral sclerosis (PLS)		27 (5.4)
Progressive muscular atrophy (PMA)		16 (3.2)
Other		1 (0.2)
Unknown		4 (0.8)
Omitted		2 (0.4)
**Ambulation status**		
Independent without assistance	N (%)	158 (31.8)
Primarily use a cane or crutches		61 (12.3)
Primarily use a walker		89 (17.7)
Primarily use a wheelchair or motorized scooter		190 (38.2)
**Feeding tube status for nutrition or hydration**		
Yes	N (%)	100 (20.1)
No		397 (79.9)
**Breathing status**		
Breathe without need for any assisted ventilation	N (%)	312 (62.8)
Non-invasive ventilation (Bipap) for <16 h in a 24 h day		139 (28.0)
Non-invasive ventilation (Bipap) for ≥16 h in a 24 h day		37 (7.4)
Tracheostomy and a ventilator attached to it		9 (1.8)
**Speech status**		
Talk clearly and no changes in speech	N (%)	174 (35.0)
Some speech changes		113 (22.7)
Impaired speech, people occasionally ask to repeat words or phrases		79 (15.9)
Impaired speech that is often not understood by others		69 (13.9)
Unable to communicate verbally		62 (12.5)
**No. of blood relatives with ALS or Frontotemporal Dementia diagnosis**		
None	N (%)	434 (87.3)
1+		33 (6.6)
2+		30 (6.0)
**Genetic test positive for mutation associated with ALS**		
Yes	N (%)	47 (9.5)
No		232 (46.7)
No genetic testing done		218 (43.9)
**States represented**		
Alabama	N (%)	5 (1.0)
Arizona		9 (1.8)
Arkansas		3 (0.6)
California		47 (9.5)
Colorado		21 (4.2)
Connecticut		3 (0.6)
Florida		38 (7.7)
Georgia		14 (2.8)
Hawaii		2 (0.4)
Idaho		5 (1.0)
Illinois		25 (5.0)
Indiana		6 (1.2)
Iowa		7 (1.4)
Kansas		3 (0.6)
Kentucky		3 (0.6)
Louisiana		8 (1.6)
Maine		3 (0.6)
Maryland		7 (1.4)
Massachusetts		7 (1.4)
Michigan		11 (2.2)
Minnesota		16 (3.2)
Missouri		9 (1.8)
Montana		2 (0.4)
Nebraska		1 (0.2)
Nevada		1 (0.2)
New Hampshire		3 (0.6)
New Jersey		16 (3.2)
New Mexico		8 (1.6)
New York		30 (6.0)
North Carolina		22 (4.4)
Ohio		24 (4.8)
Oklahoma		3 (0.6)
Oregon		11 (2.2)
Pennsylvania		18 (3.6)
Rhode Island		1 (0.2)
South Carolina		11 (2.2)
South Dakota		3 (0.6)
Tennessee		8 (1.6)
Texas		30 (6.0)
Utah		4 (0.8)
Virginia		14 (2.8)
Washington		16 (3.2)
Wisconsin		14 (2.8)
Wyoming		1 (0.2)

### Prevalence of symptomatic themes and symptoms

Of the 17 symptomatic themes, 16 had a prevalence of greater than 50% and nine had a prevalence of greater than 75% in the national cross-sectional sample (Table 2). The symptomatic themes occurring with the highest prevalence were inability to do activities, fatigue, problems with hands or fingers, and limitations with mobility or walking (Table 2). Of the 189 symptomatic questions (excluding the symptomatic themes), participants identified the following as having the highest prevalence: impaired endurance (96.1%), fatigue after physical activity (95.6%), the need for extra recovery time after activities (94.5%), the need for increased time to complete an activity (94.4%), and muscle weakness (94.1%). A listing of all 189 symptoms and their relative prevalence is available from Supplemental Table S1.

**Table 2:** Prevalence of symptomatic themes by demographic categories.

A: Theme	Full sample	Employment status			Education level			Sex			Age		
	Prevalence, %	Prevalence, %			Prevalence, %			Prevalence, %			Prevalence, %		
	All participants (N = 497)	Employed	Unable to work	p-value	College +	HS, technical, below	p-value	Male	Female	p-value	Age <63.3	Age >63.3	p-value
Problems with your hands or fingers	87.7	77.03	89.6	0.0061∗	86.49	91.34	0.16	90.94	82.35	0.007∗	88.41	87.02	0.68
Problems with your shoulders or arms	79.9	72.97	81.09	0.12	78.11	85.04	0.10	83.17	74.33	0.02	82.40	77.48	0.18
Inability to do activities	93.8	90.54	94.33	0.20	93.24	95.28	0.53	95.79	90.37	0.02	93.56	93.89	1
Hip, thigh, or knee weakness	80.1	68.92	82.03	0.0118∗	78.65	84.25	0.20	79.94	80.75	0.91	81.97	78.24	0.31
Limitations with your mobility or walking	86.7	77.03	88.42	0.01	85.68	89.76	0.29	85.11	89.84	0.17	85.84	87.40	0.69
Decreased performance in social situations	85.7	63.51	89.55	<0.0001∗	84.51	88.98	0.24	85.99	85.03	0.79	83.69	87.36	0.25
Decreased satisfaction in social situations	81.9	67.57	84.36	0.0016∗	81.03	84.25	0.50	81.17	83.42	0.55	81.97	81.61	1
Difficulty thinking	27.7	20.55	28.92	0.16	24.86	35.71	0.02	29.47	24.86	0.30	30.70	25.10	0.19
Emotional issues	68.6	71.62	68.1	0.59	67.03	73.23	0.22	66.34	72.73	0.16	75.11	62.55	0.0035∗
Impaired body image	69.6	56.76	71.8	0.01	69.11	70.87	0.74	67.53	73.26	0.19	72.10	67.05	0.24
Fatigue	92.6	89.19	93.14	0.23	91.89	94.49	0.43	91.59	94.12	0.38	93.13	91.98	0.73
Impaired sleep or daytime sleepiness	77.9	73.38	77.78	1	75.95	83.46	0.08	74.76	82.89	0.03	82.40	73.66	0.02
Pain	55.8	41.89	58.23	0.0109∗	52.17	66.40	0.0066∗	55.70	55.68	1	58.01	53.85	0.36
Gastrointestinal issues	52.7	43.24	54.33	0.10	52.34	53.54	0.84	52.94	52.46	0.93	51.32	53.64	0.65
Breathing difficulties	58.4	36.99	62.14	<0.0001∗	59.29	55.91	0.53	55.70	63.24	0.11	55.22	61.30	0.20
Choking or swallowing issues	64.0	32.88	69.36	<0.0001∗	62.67	67.72	0.34	59.61	70.97	0.0119∗	59.91	67.31	0.09
Communication difficulties	63.0	42.47	66.59	0.0001∗	61.48	67.46	0.24	60.13	67.57	0.10	61.14	64.37	0.51

B: Theme	Breathing status			Speech status			Time since first signs of weakness (months)			Feeding tube for nutrition or hydration status			Location of symptom onset		
	Prevalence, %			Prevalence, %			Prevalence, %			Prevalence, %			Prevalence, %		
	Breathe with assistive ventilation	Breathe without assistive ventilation	p-value	Able to talk clearly with no changes in speech	Others	p-value	≤34 months	>34 months	p-value	No	Yes	p-value	Limb onset	Bulbar onset	p-value
Problems with your hands or fingers	92.97	84.62	0.0068∗	85.63	88.85	0.32	82.68	92.98	0.0006∗	88.92	83.00	0.12	91.80	73.15	<0.0001∗
Problems with your shoulders or arms	87.57	75.32	0.0011∗	81.03	79.26	0.73	73.62	86.78	0.0003∗	79.35	82.00	0.68	84.13	64.81	<0.0001∗
Inability to do activities	96.76	91.99	0.04	95.98	92.57	0.17	92.13	95.45	0.14	94.46	91.00	0.24	98.15	77.78	<0.0001∗
Hip, thigh, or knee weakness	84.32	77.56	0.08	84.48	77.71	0.08	73.23	87.19	0.0001∗	80.60	78.00	0.58	86.24	59.26	<0.0001∗
Limitations with your mobility or walking	91.35	83.97	0.02	87.36	86.38	0.89	83.46	90.08	0.03	86.40	88.00	0.74	91.80	70.37	<0.0001∗
Decreased performance in social situations	93.44	81.09	0.0001∗	72.99	92.52	<0.0001∗	80.16	91.32	0.0005∗	83.08	95.96	0.0006∗	83.82	90.65	0.09
Decreased satisfaction in social situations	90.22	76.92	0.0002∗	68.97	88.82	<0.0001∗	77.87	85.95	0.02	78.79	94.00	0.0002∗	80.11	87.04	0.12
Difficulty thinking	29.05	26.86	0.60	21.05	31.23	0.02	25.60	29.96	0.31	27.74	27.37	1	30.29	19.23	0.03
Emotional issues	71.04	67.2	0.42	58.96	73.83	0.0008∗	66.80	70.83	0.38	66.08	78.79	0.02	69.15	65.42	0.48
Impaired body image	78.26	64.42	0.0012∗	65.52	71.74	0.15	61.26	78.51	<0.0001∗	68.18	75.00	0.22	72.68	59.26	0.009∗
Fatigue	96.22	90.38	0.02	89.66	94.12	0.08	93.70	91.32	0.39	91.69	96.00	0.20	92.06	93.52	0.84
Impaired sleep or daytime sleepiness	83.24	74.68	0.03	70.69	81.73	0.0064∗	77.95	77.69	1	75.82	86.00	0.03	77.25	81.48	0.43
Pain	58.24	54.34	0.45	49.71	59.06	0.06	48.21	63.90	0.0005∗	55.70	56.12	1	58.93	44.86	0.0111∗
Gastrointestinal issues	67.76	43.65	<0.0001∗	42.11	58.31	0.0006∗	49.00	56.67	0.10	48.85	68.04	0.0009∗	52.80	51.92	0.91
Breathing difficulties	89.73	39.61	<0.0001∗	35.88	70.28	<0.0001∗	52.96	64.02	0.01	51.91	84.00	<0.0001∗	53.21	75.93	<0.0001∗
Choking or swallowing issues	78.26	55.48	<0.0001∗	22.81	85.76	<0.0001∗	60.08	68.33	0.06	55.70	96.97	<0.0001∗	55.20	93.52	<0.0001∗
Communication difficulties	75.14	55.70	<0.0001∗	11.18	90.37	<0.0001∗	60.32	65.69	0.23	55.10	94.00	<0.0001∗	53.62	95.37	<0.0001∗

The p-values are from Fisher exact tests comparing responses among the subgroups.

∗Significant p-value ≤0.05 after Benjamini-Hochberg procedure was applied.

### Average life impact of symptomatic themes and symptoms

**Fig. 2:**
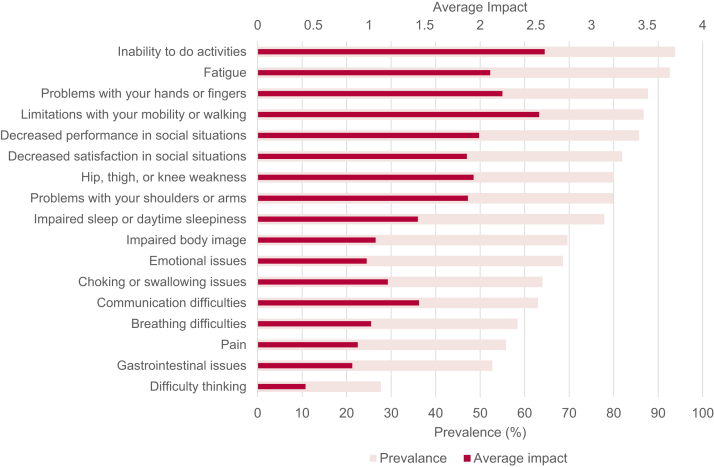
Prevalence and average impact of symptomatic themes.

Fig. 2 shows the average life impact of the symptomatic themes as compared with their prevalence. The symptomatic themes with the greatest effect on participants’ lives (highest average life impact scores) were: an inability to do activities, limitations with mobility or walking, problems with hands or fingers, and fatigue (Fig. 2). Of the 189 symptomatic questions (excluding the symptomatic themes), those that had the greatest effect on the lives of participants with ALS were: difficulty running (2.90), difficulty walking long distances (2.83), difficulty getting up from the ground (2.82), difficulty playing sports (2.73), difficulty moving quickly (2.65), difficulty walking up hills or inclines (2.62), and difficulty going upstairs (2.62). A listing of all 189 symptoms and their relative average life impact score is available from Supplemental Table S1.

### Population impact scores

The symptomatic themes with the greatest population impact scores (0–4) were: an inability to do activities (2.42), limitations with mobility or walking (2.19), fatigue (1.94), problems with hands or fingers (1.93), and decreased performance in social situations (1.7). The symptomatic questions, excluding the symptomatic themes, with the greatest population impact scores were: difficulty running (2.71), difficulty getting up from the floor or ground (2.63), difficulty walking long distances (2.56), difficulty playing sports (2.51), impaired endurance (2.45), and difficulty moving quickly (2.41). A listing of all 189 symptoms and their relative population impact score is available in Supplemental Table S1.

### Subgroup analysis in prevalence of symptomatic themes

There were differences in the prevalence of numerous symptomatic themes based on subgroup analysis. Eight of the 17 symptomatic themes were less prevalent among employed individuals relative to unemployed individuals (Table 2). The largest difference in prevalence was seen in the theme focused on choking or swallowing issues, with employed individuals having a prevalence of 32.9% compared to unemployed individuals having a prevalence of 69.4% (p < 0.0001).

Breathing status was associated with differences in the prevalence of nine of the 17 symptomatic themes, with those requiring assistive ventilation reporting greater prevalence of all symptomatic themes compared to those not requiring assistive ventilation (Table 2).

Speech status was associated with differences in the prevalence of eight symptomatic themes, including decreased performance in social situations (p < 0.0001), decreased satisfaction in social situations (p < 0.0001), emotional issues (p = 0.0008), impaired sleep or daytime sleepiness (p = 0.0064), gastrointestinal issues (p = 0.0006), breathing difficulties (p < 0.0001), choking or swallowing issues (p < 0.0001), and communication difficulties (p < 0.0001) (Table 2). These eight symptomatic themes were more prevalent in individuals experiencing difficulty speaking and/or changes in speech experienced relative to individuals experiencing no changes in speech.

Use of a feeding tube for nutrition or hydration was associated with six symptomatic themes, including decreased performance in social situations (p = 0.0006), decreased satisfaction in social situations (p = 0.0002), gastrointestinal issues (p = 0.0009), breathing difficulties (p < 0.0001), choking or swallowing issues (p < 0.0001), and communication difficulties (p < 0.0001) (Table 2). These six symptomatic themes were all more prevalent in individuals requiring a feeding tube for nutrition and/or hydration relative to individuals who didn't require a feeding tube.

Six of the 17 symptomatic themes were more prevalent in individuals with above the mean values of amount of time since noticing first signs of weakness compared to those with values below the mean (Table 2). The largest difference in prevalence was seen in impaired body image, with individuals experiencing a longer time since first signs of weakness having a prevalence of 78.5% compared to those with a more recent experience of first signs of weakness having a prevalence of 61.3% (p < 0.0001). The second largest difference in prevalence was seen in pain, with individuals with a longer time since first signs of weakness having a prevalence of 63.9% compared to those with a more recent experience of first signs of weakness having a prevalence of 48.2% (p = 0.0005).

Seven of the 17 symptomatic themes were more prevalent in those with limb symptom onset relative to those with bulbar onset (Table 2). These symptomatic themes included problems with hands or fingers (p < 0.0001), problems with shoulders or arms (p < 0.0001), inability to do activities (p < 0.0001), hip, thigh or knee weakness (p < 0.0001), limitations with mobility (p < 0.0001), impaired body image (p = 0.009), and pain (p = 0.0111). Three of the 17 symptomatic themes were more prevalent in those with bulbar symptom onset compared to those with limb onset, including breathing difficulties (p < 0.0001), choking or swallowing issues (p < 0.0001), and communication difficulties (p < 0.0001). The largest difference in prevalence was seen in communication difficulties, where individuals with bulbar onset had a prevalence of 95.4% and those with limb onset had a prevalence of 53.6% (p < 0.0001).

In examining differences between ALS type, most participants reported diagnoses of ALS (n = 434), with the remaining participant diagnoses consisting of ALS with frontotemporal dementia (n = 10), primary lateral sclerosis (n = 27), progressive muscular atrophy (n = 16), Kennedy's disease (n = 3), other (n = 1), and unknown (n = 4). In Table 3, we examine the difference between ALS type. No differences between groups were found to be statistically significant.

**Table 3:** Prevalence of symptomatic themes by ALS type.

Theme	Prevalence, %				p-value		
	ALS (n = 434)	PLS (n = 27)	PMA (n = 16)	ALS with frontotemporal dementia (n = 10)	ALS vs. PLS	ALS vs. PMA	ALS vs. ALS with frontotemporal dementia
Problems with your hands or fingers	89.17	85.19	75.00	60	0.52	0.10	0.02
Problems with your shoulders or arms	81.57	77.78	62.50	50	0.61	0.10	0.03
Inability to do activities	93.78	100	100	70	0.39	0.61	0.02
Hip, thigh, or knee weakness	80.88	85.19	81.25	50	0.80	1	0.03
Limitations with your mobility or walking	86.87	100	81.25	70	0.04	0.46	0.14
Decreased performance in social situations	85.65	92.59	75.00	80	0.40	0.27	0.64
Decreased satisfaction in social situations	81.99	88.89	75.00	70	0.45	0.51	0.40
Difficulty thinking	25.76	38.46	40.00	60	0.17	0.24	0.03
Emotional issues	67.67	80.77	66.67	80	0.20	1	0.51
Impaired body image	69.05	70.37	93.75	70	1	0.05	1
Fatigue	93.09	92.59	81.25	90	0.71	0.10	0.52
Impaired sleep or daytime sleepiness	78.11	77.78	81.25	60	1	1	0.24
Pain	54.88	70.37	43.75	60	0.16	0.45	1
Gastrointestinal issues	52.46	55.56	37.50	70	0.84	0.31	0.35
Breathing difficulties	59.77	48.15	43.75	40	0.31	0.21	0.33
Choking or swallowing issues	64.27	70.37	43.75	60	0.68	0.11	0.75
Communication difficulties	62.70	77.78	50.00	60	0.15	0.31	1

The p-values are from Fisher exact tests comparing responses among the subgroups.

## Discussion

This research represents one of the largest studies evaluating patient-reported disease burden in ALS. Our study describes the many symptoms and symptomatic themes that generate disease burden in individuals with ALS. These symptoms have a variable prevalence and relative importance in this population. In this study, we demonstrate that individuals with ALS are significantly impacted by symptoms representing physical, emotional, and social health.

Here, we document results from one of the largest national ALS studies designed to identify the phenotypic profile and relative importance of individual symptoms in a sample of individuals with ALS. Our study provides researchers with necessary and extensive baseline data to help plan future therapeutic trials focused on the issues that matter most to individuals with ALS. Interestingly, many of the symptomatic themes that were identified by participants as having a high importance would not be measured by commonly used ALS outcome measures such as the ALSFRS-R.^12^ Concepts such as fatigue, emotional and social health, impaired sleep and daytime sleepiness, and pain are all important to ALS patients and would be worth monitoring during future interventional studies.

We found that many symptomatic themes have a high prevalence in the ALS population. An inability to do activities, fatigue, problems with hands or fingers, and limitations with mobility or walking were some of the most prevalent symptomatic themes in this sample of individuals with ALS. We found that the most prevalent symptomatic theme, the inability to do activities, was also the symptomatic theme with the greatest population impact.

Results from subgroup analysis provide insight into how symptom prevalence differs depending on patient characteristics. The demographic features associated with the most widespread variation in symptomatic theme prevalence were related to participant breathing status and location of symptom onset. As expected, those requiring assisted ventilation to breathe had a higher prevalence in the majority of symptomatic themes, with nine of the 17 themes significantly varying based on breathing status. Not surprisingly, for those with bulbar onset, individuals had a higher prevalence of symptoms associated with breathing difficulties, choking or swallowing issues, and communication difficulties. For those with limb onset, individuals had a higher prevalence of symptoms in themes associated with limb strength (i.e., problems with hands or fingers, problems with shoulders or arms, inability to do activities, hip, thigh or knee weakness, limitations with mobility or walking, and impaired body image). Employment status displayed variation in disease burden, with eight symptomatic themes varying in prevalence. This suggests, perhaps unsurprisingly, that those with a greater disease burden are less likely to have employment. This also may be a marker of early versus late disease status. Similarly, impaired speech and requiring a feeding tube were associated with a greater prevalence in eight and six of the 17 symptomatic themes respectively.

Given the varying clinical courses between different ALS types (e.g., ALS relative to PLS), it is reasonable to expect differences in prevalence of symptoms across ALS types. While differences between ALS type (ALS vs. PLS, ALS vs. PMA, and ALS vs. ALS with frontotemporal dementia) were not found to be statistically significant in our study, we did find differences in magnitudes of symptom prevalence across ALS types. For example, we found a difference in the prevalence of difficulty thinking between ALS (25.76%) and ALS with frontotemporal dementia (60%). We also found a difference in prevalence of pain between ALS (54.88%) and PLS (70.37%). We suspect that these differences did not have a significant p value in part due to the small sample size of each of these subgroups.

We acknowledge that our sample may not be representative of the entire population of individuals with ALS. Participants for the cross-sectional study were required to be members of the CDC's National ALS Registry. It is likely that those without access to the internet because of socioeconomic factors were underrepresented in our sample. Individuals with ALS who were too sick to participate and those not interested in clinical research were likely not included in our sample. This study was limited to the United States, and, therefore, results must be interpreted in this context. Lastly, this registry includes several ALS variants (e.g. PMA, PLS, ALS with frontotemporal dementia), which have distinct phenotypical presentations.

Of note, three participants reported that they had Kennedy's disease (Spinal Bulbar Muscular Atrophy) during our cross-sectional study. All three participants would have been required to report that they have ALS to join the registry. As Kennedy's disease can often be misdiagnosed as ALS initially, we suspect that these participants were initially misdiagnosed with ALS prior to their diagnosis of Kennedy's disease. Given our predetermined protocol, their responses were retained in our analysis.

This research took extensive steps to identify the most important and common symptoms in ALS. Fifteen individuals were initially interviewed to identify potential symptoms of importance. Although this is a relatively small number, concept saturation was reached during these the last of these interviews and participants in our cross-sectional study were given the opportunity to identify symptoms otherwise not identified during the initial interviews. As we have seen in other neurological conditions, we believe that this methodology is sufficient to identify the major symptoms associated with ALS.^6^, ^7^, ^8^, ^9^, ^10^, ^11^

Many registry members who were sent an email with a link to our survey did not respond. Although the survey was first sent to 11,373 and then to 11,836 registry members, it's likely that many of those members were no longer active registry members. Registry members may have passed away, may have progressed too far in their disease to answer survey questions, or had a change of email address. For others, the length of the survey may have been prohibitive. While 497 participants completed the demographics section and answered at least one question, only 447 participants responded to the last survey question. This drop from 497 to 447 participant responses suggests a 10% attrition rate. Missing responses appear to have occurred non-randomly, with more missing responses later in the survey. This suggests that the high volume of questions in the survey may have led some participants to become fatigued with the survey length and not answer questions later in the survey. Furthermore, it was not possible to track the number of times participants self-enrolled due to the anonymous nature of the online survey. Participants may have completed the survey more than once, despite being asked to complete the survey only once.

Patient Reported Impact of Symptoms in Amyotrophic Lateral Sclerosis (PRISM-ALS) adds to the available knowledge regarding the multifactorial symptomatic burden that individuals with ALS experience. Similar to other PRISM studies, this study shows that, despite some overlap, the specific symptoms and impact of symptoms vary based on the specific neuromuscular disease population.^6^, ^7^, ^8^, ^9^, ^10^, ^11^ In addition to the many commonly recognized symptoms regarding physical dysfunction, participants identified a high prevalence of issues in other areas including impaired sleep or daytime sleepiness, gastrointestinal issues, and difficulty thinking. These particular issues are typically less focused on by treating physicians yet represent areas of potential therapeutic interventions. An understanding of the prevalence and relative impact of these symptoms and symptomatic themes is beneficial for those caring for this population. This understanding is also important for the planning and implementation of future ALS therapeutic trials that seek to reduce the symptomatic burden in this population and the development, validation, and selection of clinical trial outcome measures that are capable of measuring what is most important to patients with ALS.

## Contributors

CZ performed the investigation, handled project administration, analyzed and interpreted the data, and drafted the manuscript including all tables and figures. CH designed and conceptualized the study, analyzed and interpreted the data, and revised the manuscript for intellectual content. JS, SR, DA, EW, JSW, and AV revised the manuscript. ND and JH analyzed the data. JW, JC, RB, VG, and JMS participated in an expert review of the study. PM and MB participated in an expert review of the study and assisted in the acquisition of data. All authors had full access to study data and take responsibility for the integrity of the data and accuracy of the data analysis. All authors had final responsibility for the decision to submit for publication.

## Data sharing statement

Anonymized data will be shared by reasonable request from qualified investigators.

## Declaration of interests

CZ has previously provided consultation to Recursion Pharmaceuticals and reports no other disclosures. RB receives grants or contracts from the 10.13039/100000971ALS Association, Medicinova, Healey Center, and Orion with payments through 10.13039/100006510Duke University for various ALS projects and ALS trials. He receives book royalities through Demos Publishing. He provides consultation to ALS Association, Alexion, Amylyx, Apellis, Black Swan, Corcept, Cytokinetics, GenieUS, Guidepoint, ITF Pharma, New Biotic, Shinkei, and Woolsey Pharma. He has received payments for speaking on ALS from Amylyx. He has received payments for developing education materials on ALS from Projects in Knowledge and Medscape. He has participated on Data Safety Monitoring Boards for AB Science, Biogen, Brainstorm, Neurosense, and PTC. He has served on the scientific advisory board at GenieUS. VG is an employee of Biohaven Pharmaceuticals (his contribution to this work was done as part of his role at the University of Miami). He has received consultation/advisory board fees from Amylyx Pharmacuticals. JS has received grants or contracts from 10.13039/100000065NINDS (U01 ReSolve U01NS101944), NINDS (R21 CAPN3 R21TR003184), FSHD Canada (MOVE + FSHD), FSHD Society (MOVE FSHD), 10.13039/100004952MDA (MD CRNG), 10.13039/100012644Friends of FSH Research (MOVE FSHD). He has provided consultation and served on SAB with Avidity, Dyne, Fulcrum, Arrowhead, ML Bio, and Sarepta. He has provided consultation to MT Pharma, He has provided consultation and served on the advisory board for Amylyx. He has served on the steering committee at Roche. MB reports grants from 10.13039/100000971ALS Association (16-TACL-242 and 17-LGCA-331), 10.13039/100000002NIH (U54-NS092091, R01-NS105479 and U01- NS107027), and 10.13039/100005202Muscular Dystrophy Association (645863). He has received consulting fees from Alector, Biogen, Novartis, Sanofi, UCB, UniQure. The University of Miami has licensed intellectual property to Biogen to support design of the ATLAS study (UMIP142-A.) In addition, he has a provisional patent pending entitled ‘Determining Onset of Amyotrophic Lateral Sclerosis.’ He serves on the Board of Trustees for the ALS Association (unpaid). CH receives royalty payments through the University of Rochester for copyrighted patient reported outcome measures including one for ALS. He has provided consultation to Biogen Idec, Ionis Pharmaceuticals, aTyr Pharma, AMO Pharma, Acceleron Pharma, Cytokinetics, Expansion Therapeutics, Harmony Biosciences, Regeneron Pharmaceuticals, Astellas Pharmaceuticals, AveXis, Recursion Pharmaceuticals, IRIS Medicine, Inc., Takeda Pharmaceutical Company, Scholar Rock, Avidity Biosciences, Novartis Pharmaceuticals Corporation, SwanBio Therapeutics, Neurocrine Biosciences, and the Marigold Foundation. He is scheduled to have travel reimbursement to present at the upcoming Muscle Study Group Meeting. JS, SR, DA, EW, JSW, AV, ND, JH, JW, JC, and PM report no disclosures.

## Appendix A Supplementary data

Supplemental Phase 1 Interview Guide

Supplementary Table S1

STROBE_checklist_cross-sectional
