# Supplementary material for: Patient reported impact of symptoms in amyotrophic lateral sclerosis (PRISM-ALS): A national, cross-sectional study
Source: eClinicalMedicine. 2022 Dec 13;55:101768. doi: 10.1016/j.eclinm.2022.101768 (PMC9755057; doi:10.1016/j.eclinm.2022.101768)
Supplement: Supplemental Phase 1 Interview Guide [file mmc1.pdf]

Appendix 1: A list of interview questions for ALS patients

1) Which symptoms of ALS have the greatest impact on a person's quality-of-life and disease burden?
2) How is an individual's life altered by having ALS?
3) What changes would greatly improve your overall satisfaction with life?

Physical

4) Could you give examples of how a person with ALS is affected physically by the disease?
5) Has pain or fatigue interfered with your life?
6) Has weakness or difficulty walking interfered with your life?
7) What type of physical function limitations are most important to a patient with ALS?
8) Do you have problems with your swallowing or eating?
9) Do you experience weakness at your hands or arms? How does this affect your life?

9a) What other activities do you now have difficulty doing?

Mental

10) Do you have difficulty concentrating, reading, remembering, or thinking? How has this affected you?

Emotional

11) Could you give examples of how a person with ALS is affected emotionally by the disease?

Social

12) How does ALS affect the social health of a person? (e.g. their relationship with family, work colleagues, or friends)
13) What ways does ALS affect family life?
14) Has ALS interfered with how you interact with others?
15) What activities with your friends do you have difficulty participating in?
16) How has ALS interfered with how you communicate with others?

Everyday

17) What is the most troubling problem a person with ALS has to face?
18) What are the "little-things" that are affected by and important to people with ALS?
19) What are the most important questions a healthcare provider should ask someone with ALS?
20) What are the ways that ALS affects someone's everyday life?
21) What areas of your life would you most like to improve?
22) How does ALS interfere with your job or your ability to get a job?
23) What type of activities around the house do you have difficulty with?
24) What ways is a patient's independence affected by ALS?
25) Has ALS changed how you view yourself or your perceived body image?
26) What areas of your life would you most like to improve?
27) Do you have any problems with your sleep or with daytime sleepiness?
28) Do you have problems breathing? If so how does this affect you?
29) Do cramps or fasciculations affect your life? How about spasticity of the limbs?
30) How is your bladder and bowel function? Does this interfere with your life?
31) If a successful therapy was developed, what would it do for you?
32) Are there other areas that have a major impact on your quality-of-life or disease burden that we did not mention?
