# Supplementary material for: Patient reported impact of symptoms in amyotrophic lateral sclerosis (PRISM-ALS): A national, cross-sectional study
Source: eClinicalMedicine. 2022 Dec 13;55:101768. doi: 10.1016/j.eclinm.2022.101768 (PMC9755057; doi:10.1016/j.eclinm.2022.101768)
Supplement: Supplementary Table S1 [file mmc2.docx]

|  |  |  |  |  |  |  |  |  |  |
| --- | --- | --- | --- | --- | --- | --- | --- | --- | --- |
|  | **Question number** | **Symptom** | **No. of Responses** | **No. Experiencing** | **Prevalence (%)** | **Average life impact score** | **SD** | **Population impact score** |  |
|  | q1a | Problems with your hands or fingers | 497 | 436 | 87.7 | 2.2 | 1.37 | 1.93 |  |
|  | q1b | Problems with your shoulders or arms | 497 | 397 | 79.9 | 1.89 | 1.42 | 1.51 |  |
|  | q1c | Inability to do activities | 497 | 466 | 93.8 | 2.58 | 1.23 | 2.42 |  |
|  | q1d | Hip, thigh, or knee weakness | 497 | 398 | 80.1 | 1.94 | 1.43 | 1.55 |  |
|  | q1e | Limitations with your mobility or walking | 497 | 431 | 86.7 | 2.53 | 1.41 | 2.19 |  |
|  | q1f | Decreased performance in social situations | 495 | 424 | 85.7 | 1.99 | 1.37 | 1.7 |  |
|  | q1g | Decreased satisfaction in social situations | 496 | 406 | 81.9 | 1.88 | 1.38 | 1.53 |  |
|  | q1h | Difficulty thinking | 488 | 135 | 27.7 | 0.43 | 0.9 | 0.12 |  |
|  | q1i | Emotional issues | 494 | 339 | 68.6 | 0.98 | 1.2 | 0.67 |  |
|  | q1j | Impaired body image | 496 | 345 | 69.6 | 1.06 | 1.23 | 0.74 |  |
|  | q1k | Fatigue | 497 | 460 | 92.6 | 2.09 | 1.19 | 1.94 |  |
|  | q1l | Impaired sleep or daytime sleepiness | 497 | 387 | 77.9 | 1.44 | 1.23 | 1.12 |  |
|  | q1m | Pain | 493 | 275 | 55.8 | 0.9 | 1.19 | 0.5 |  |
|  | q1n | Gastrointestinal issues | 490 | 258 | 52.7 | 0.85 | 1.17 | 0.45 |  |
|  | q1o | Breathing difficulties | 493 | 288 | 58.4 | 1.02 | 1.31 | 0.6 |  |
|  | q1p | Choking or swallowing issues | 494 | 316 | 64 | 1.17 | 1.34 | 0.75 |  |
|  | q1q | Communication difficulties | 492 | 310 | 63 | 1.45 | 1.52 | 0.91 |  |
|  | q2a | Hand weakness | 491 | 433 | 88.2 | 2.29 | 1.37 | 2.02 |  |
|  | q2b | Difficulty opening jars or bottles | 491 | 441 | 89.8 | 2.39 | 1.39 | 2.15 |  |
|  | q2c | Finger weakness | 491 | 431 | 87.8 | 2.3 | 1.4 | 2.01 |  |
|  | q2d | Dropping objects with your hands | 489 | 403 | 82.4 | 2.04 | 1.43 | 1.68 |  |
|  | q2e | Difficulty using a pen or pencil | 491 | 398 | 81.1 | 2.02 | 1.5 | 1.64 |  |
|  | q2f | Difficulty using a spoon or fork | 491 | 394 | 80.2 | 1.93 | 1.46 | 1.55 |  |
|  | q2g | Difficulty doing things with your hands | 491 | 420 | 85.5 | 2.18 | 1.42 | 1.87 |  |
|  | q2h | Difficulty washing your hair | 490 | 358 | 73.1 | 1.86 | 1.62 | 1.36 |  |
|  | q2i | Difficulty picking things up with your hands | 490 | 406 | 82.9 | 2.04 | 1.48 | 1.69 |  |
|  | q2j | Difficulty using buttons or zippers | 490 | 418 | 85.3 | 2.24 | 1.52 | 1.92 |  |
|  | q2k | Problems cutting food | 488 | 395 | 80.9 | 2.07 | 1.55 | 1.68 |  |
|  | q3a | Arm weakness | 489 | 426 | 87.1 | 2.08 | 1.39 | 1.81 |  |
|  | q3b | Difficulty lifting objects | 489 | 446 | 91.2 | 2.33 | 1.32 | 2.13 |  |
|  | q3c | Shoulder weakness | 488 | 396 | 81.1 | 1.9 | 1.42 | 1.54 |  |
|  | q3d | Decreased ability to carry a heavy load with your arms | 489 | 457 | 93.5 | 2.55 | 1.32 | 2.38 |  |
|  | q3e | Neck weakness | 486 | 314 | 64.6 | 1.27 | 1.37 | 0.82 |  |
|  | q3f | Difficulty reaching for objects overhead | 489 | 416 | 85.1 | 2.23 | 1.5 | 1.9 |  |
|  | q3g | Muscle weakness | 489 | 460 | 94.1 | 2.56 | 1.27 | 2.41 |  |
|  | q3h | Difficulty pushing yourself up with your arms | 489 | 426 | 87.1 | 2.29 | 1.4 | 1.99 |  |
|  | q3i | Impaired coordination | 488 | 404 | 82.8 | 1.97 | 1.39 | 1.63 |  |
|  | q4a | Difficulty maintaining your personal hygiene | 482 | 373 | 77.4 | 1.81 | 1.53 | 1.4 |  |
|  | q4b | Difficulty feeding yourself | 481 | 313 | 65.1 | 1.44 | 1.55 | 0.93 |  |
|  | q4c | The need for increased time to complete an activity | 482 | 455 | 94.4 | 2.24 | 1.31 | 2.11 |  |
|  | q4d | Difficulty writing on a piece of paper | 482 | 383 | 79.5 | 1.91 | 1.54 | 1.52 |  |
|  | q4e | Impaired ability to exercise | 482 | 451 | 93.6 | 2.49 | 1.35 | 2.33 |  |
|  | q4f | Difficulty operating a motor vehicle | 479 | 343 | 71.6 | 2.07 | 1.74 | 1.48 |  |
|  | q4g | Trouble scratching yourself | 480 | 320 | 66.7 | 1.57 | 1.58 | 1.05 |  |
|  | q4h | Difficulty using a keyboard | 480 | 332 | 69.2 | 1.56 | 1.51 | 1.08 |  |
|  | q4i | Difficulty cleaning the house | 478 | 408 | 85.4 | 2.37 | 1.5 | 2.02 |  |
|  | q4j | Difficulty washing the dishes | 477 | 385 | 80.7 | 2.14 | 1.57 | 1.73 |  |
|  | q4k | Difficulty preparing food | 479 | 398 | 83.1 | 2.23 | 1.55 | 1.85 |  |
|  | q4l | Difficulty dressing yourself | 482 | 417 | 86.5 | 2.21 | 1.49 | 1.91 |  |
|  | q4m | Difficulty using a computer tablet | 476 | 299 | 62.8 | 1.38 | 1.46 | 0.86 |  |
|  | q4n | Difficulty drinking from a glass | 480 | 340 | 70.8 | 1.53 | 1.54 | 1.09 |  |
|  | q4o | Difficulty changing lightbulbs | 469 | 350 | 74.6 | 1.91 | 1.71 | 1.43 |  |
|  | q4p | Difficulty cutting nails | 481 | 406 | 84.4 | 2.18 | 1.57 | 1.84 |  |
|  | q4q | Difficulty getting on and off a toilet | 481 | 363 | 75.5 | 1.83 | 1.61 | 1.38 |  |
|  | q4r | Trouble getting in or out of a tub or shower | 481 | 372 | 77.3 | 1.98 | 1.63 | 1.53 |  |
|  | q4s | Difficulty turning pages | 480 | 321 | 66.9 | 1.53 | 1.54 | 1.02 |  |
|  | q4t | Difficulty dancing | 460 | 372 | 80.9 | 2.24 | 1.72 | 1.81 |  |
|  | q4u | Difficulty playing sports | 468 | 431 | 92.1 | 2.73 | 1.51 | 2.51 |  |
|  | q4v | Difficulty putting on your shoes | 481 | 408 | 84.8 | 2.2 | 1.49 | 1.86 |  |
|  | q4w | Difficulty shaking hands | 479 | 269 | 56.2 | 1.26 | 1.55 | 0.71 |  |
|  | q4x | Difficulty opening and closing doors | 481 | 343 | 71.3 | 1.6 | 1.54 | 1.14 |  |
|  | q4y | Difficulty dressing your lower body (pants) | 481 | 413 | 85.9 | 2.17 | 1.53 | 1.87 |  |
|  | q4z | Difficulty dressing your upper body (shirts) | 481 | 392 | 81.5 | 2.03 | 1.52 | 1.66 |  |
|  | q4aa | Difficulty getting in and out of vehicles | 481 | 413 | 85.9 | 2.03 | 1.53 | 1.74 |  |
|  | q5a | Leg weakness | 475 | 407 | 85.7 | 2.39 | 1.46 | 2.05 |  |
|  | q5b | Foot drop | 474 | 329 | 69.4 | 1.96 | 1.64 | 1.36 |  |
|  | q5c | Difficulty getting up from the floor or ground | 475 | 443 | 93.3 | 2.82 | 1.42 | 2.63 |  |
|  | q5d | Difficulty rising from a seated position | 475 | 409 | 86.1 | 2.24 | 1.45 | 1.93 |  |
|  | q5e | Leg stiffness | 474 | 354 | 74.7 | 1.76 | 1.53 | 1.31 |  |
|  | q5f | Ankle weakness | 474 | 337 | 71.1 | 1.73 | 1.58 | 1.23 |  |
|  | q5g | Difficulty standing | 476 | 382 | 80.3 | 2.09 | 1.56 | 1.68 |  |
|  | q6a | Impaired walking | 472 | 399 | 84.5 | 2.54 | 1.51 | 2.15 |  |
|  | q6b | Falls | 471 | 343 | 72.8 | 1.94 | 1.59 | 1.41 |  |
|  | q6c | Difficulty walking long distances | 469 | 424 | 90.4 | 2.83 | 1.44 | 2.56 |  |
|  | q6d | Difficulty with your balance | 470 | 405 | 86.2 | 2.38 | 1.54 | 2.05 |  |
|  | q6e | Difficulty going upstairs | 468 | 410 | 87.6 | 2.62 | 1.52 | 2.29 |  |
|  | q6f | Having to hold on to things when walking | 467 | 380 | 81.4 | 2.34 | 1.63 | 1.91 |  |
|  | q6g | Fear of falling | 471 | 375 | 79.6 | 2.28 | 1.63 | 1.81 |  |
|  | q6h | Difficulty moving quickly | 471 | 428 | 90.9 | 2.65 | 1.49 | 2.41 |  |
|  | q6i | Difficulty running | 465 | 435 | 93.5 | 2.9 | 1.52 | 2.71 |  |
|  | q6j | Tripping | 470 | 376 | 80 | 2.2 | 1.61 | 1.76 |  |
|  | q6k | Difficulty going downstairs | 469 | 381 | 81.2 | 2.39 | 1.62 | 1.94 |  |
|  | q6l | Difficulty getting up quickly | 471 | 416 | 88.3 | 2.51 | 1.56 | 2.22 |  |
|  | q6m | Difficulty walking up hills or inclines | 468 | 422 | 90.2 | 2.62 | 1.47 | 2.36 |  |
|  | q7a | Decreased independence | 467 | 425 | 91 | 2.61 | 1.4 | 2.37 |  |
|  | q7b | Reliance on family members | 465 | 421 | 90.5 | 2.57 | 1.43 | 2.33 |  |
|  | q7c | Inability to go out | 467 | 347 | 74.3 | 1.92 | 1.54 | 1.43 |  |
|  | q7d | Impaired sexual function | 434 | 309 | 71.2 | 1.95 | 1.65 | 1.39 |  |
|  | q7e | Difficulty holding children | 452 | 327 | 72.3 | 1.93 | 1.64 | 1.39 |  |
|  | q7f | Difficulty having a romantic relationship | 444 | 274 | 61.7 | 1.6 | 1.63 | 0.99 |  |
|  | q7g | Social interaction limited by eating difficulties | 467 | 336 | 71.9 | 1.63 | 1.55 | 1.18 |  |
|  | q7h | Difficulty helping with family chores | 464 | 416 | 89.7 | 2.53 | 1.46 | 2.27 |  |
|  | q7i | Reliance on friends | 466 | 372 | 79.8 | 1.99 | 1.49 | 1.59 |  |
|  | q7j | Difficulty keeping up with friends | 464 | 370 | 79.7 | 1.88 | 1.49 | 1.5 |  |
|  | q8a | Social isolation | 464 | 308 | 66.4 | 1.56 | 1.42 | 1.04 |  |
|  | q8b | Impaired interactions with friends | 464 | 331 | 71.3 | 1.59 | 1.4 | 1.13 |  |
|  | q8c | Inability to participate in fun activities | 463 | 393 | 84.9 | 2.22 | 1.45 | 1.88 |  |
|  | q8d | Impaired interaction with family members | 464 | 322 | 69.4 | 1.53 | 1.44 | 1.06 |  |
|  | q8e | Feeling excluded | 462 | 268 | 58 | 1.28 | 1.47 | 0.74 |  |
|  | q8f | Dissatisfied with social interactions | 462 | 293 | 63.4 | 1.32 | 1.43 | 0.84 |  |
|  | q8g | Difficulty getting around in large crowds | 459 | 357 | 77.8 | 1.83 | 1.52 | 1.43 |  |
|  | q9a | Problems concentrating | 459 | 171 | 37.3 | 0.61 | 1.04 | 0.23 |  |
|  | q9b | Memory deficits | 459 | 160 | 34.9 | 0.54 | 1.02 | 0.19 |  |
|  | q9c | Impaired short term memory | 460 | 157 | 34.1 | 0.53 | 1.01 | 0.18 |  |
|  | q9d | Difficulty reading | 457 | 110 | 24.1 | 0.42 | 0.96 | 0.1 |  |
|  | q9e | Difficulty recalling words | 459 | 188 | 41 | 0.55 | 1.03 | 0.23 |  |
|  | q9f | Forgetfulness | 458 | 177 | 38.6 | 0.51 | 0.99 | 0.2 |  |
|  | q9g | Learning difficulties | 459 | 95 | 20.7 | 0.3 | 0.82 | 0.06 |  |
|  | q9h | Difficulty with comprehension | 457 | 91 | 19.9 | 0.32 | 0.81 | 0.06 |  |
|  | q10a | Frustration | 459 | 417 | 90.8 | 1.92 | 1.32 | 1.75 |  |
|  | q10b | Anxiety | 458 | 341 | 74.5 | 1.37 | 1.36 | 1.02 |  |
|  | q10c | Fear | 456 | 327 | 71.7 | 1.27 | 1.35 | 0.91 |  |
|  | q10d | Embarrassment | 457 | 334 | 73.1 | 1.23 | 1.31 | 0.9 |  |
|  | q10e | Anger | 458 | 304 | 66.4 | 1.03 | 1.28 | 0.69 |  |
|  | q10f | Agitation | 458 | 298 | 65.1 | 1.06 | 1.25 | 0.69 |  |
|  | q10g | Emotional strain | 458 | 350 | 76.4 | 1.34 | 1.33 | 1.02 |  |
|  | q10h | A feeling of helplessness | 458 | 366 | 79.9 | 1.7 | 1.45 | 1.36 |  |
|  | q10i | Fear of disease progression | 457 | 397 | 86.9 | 1.84 | 1.49 | 1.6 |  |
|  | q10j | Stress | 459 | 380 | 82.8 | 1.49 | 1.35 | 1.23 |  |
|  | q10k | A feeling of loss of control | 459 | 399 | 86.9 | 1.89 | 1.44 | 1.64 |  |
|  | q10l | Moodiness | 458 | 322 | 70.3 | 1.1 | 1.26 | 0.77 |  |
|  | q10m | Decreased motivation | 457 | 343 | 75.1 | 1.34 | 1.32 | 1 |  |
|  | q10n | Reduced enjoyment with activities | 458 | 369 | 80.6 | 1.58 | 1.35 | 1.27 |  |
|  | q10o | Sadness | 459 | 350 | 76.3 | 1.31 | 1.34 | 1 |  |
|  | q10p | Feeling like a burden to family and friends | 458 | 381 | 83.2 | 1.93 | 1.43 | 1.61 |  |
|  | q10q | Difficulty controlling your laughter or crying | 458 | 217 | 47.4 | 0.85 | 1.22 | 0.4 |  |
|  | q10r | Depression | 457 | 264 | 57.8 | 0.93 | 1.26 | 0.54 |  |
|  | q11a | Weight loss | 458 | 235 | 51.3 | 0.83 | 1.25 | 0.42 |  |
|  | q11b | Loss of muscle | 458 | 430 | 93.9 | 2.45 | 1.33 | 2.3 |  |
|  | q11c | Runny nose | 456 | 268 | 58.8 | 0.95 | 1.25 | 0.56 |  |
|  | q11d | Embarrassed about how you sound | 455 | 236 | 51.9 | 0.94 | 1.33 | 0.49 |  |
|  | q11e | Fasciculations | 445 | 360 | 80.9 | 1.09 | 1.3 | 0.89 |  |
|  | q11f | Muscle twitching | 458 | 399 | 87.1 | 1.2 | 1.34 | 1.05 |  |
|  | q11g | Excessive sweating | 455 | 134 | 29.5 | 0.42 | 0.95 | 0.12 |  |
|  | q12a | Tired muscles | 458 | 426 | 93 | 2.28 | 1.27 | 2.12 |  |
|  | q12b | The need for extra recovery time after activities | 457 | 432 | 94.5 | 2.34 | 1.26 | 2.21 |  |
|  | q12c | Fatigue after physical activity | 457 | 437 | 95.6 | 2.43 | 1.26 | 2.33 |  |
|  | q12d | Impaired endurance | 457 | 439 | 96.1 | 2.55 | 1.25 | 2.45 |  |
|  | q12e | Muscle fatigue | 458 | 429 | 93.7 | 2.45 | 1.28 | 2.29 |  |
|  | q13a | Daytime sleepiness | 457 | 353 | 77.2 | 1.34 | 1.19 | 1.03 |  |
|  | q13b | Restless legs | 457 | 207 | 45.3 | 0.83 | 1.24 | 0.37 |  |
|  | q13c | Insomnia | 456 | 198 | 43.4 | 0.78 | 1.21 | 0.34 |  |
|  | q13d | Difficulty staying asleep | 456 | 270 | 59.2 | 0.98 | 1.24 | 0.58 |  |
|  | q13e | Waking up for long periods of time during the night | 456 | 203 | 44.5 | 0.77 | 1.23 | 0.34 |  |
|  | q13f | Difficulty changing positions while in bed | 457 | 351 | 76.8 | 1.83 | 1.56 | 1.41 |  |
|  | q13g | Having to sleep a lot | 457 | 257 | 56.2 | 0.97 | 1.26 | 0.55 |  |
|  | q13h | Difficulty falling asleep at night | 455 | 182 | 40 | 0.67 | 1.17 | 0.27 |  |
|  | q14a | Muscle cramps | 456 | 367 | 80.5 | 1.41 | 1.31 | 1.14 |  |
|  | q14b | Neck pain | 456 | 276 | 60.5 | 1.08 | 1.32 | 0.65 |  |
|  | q14c | Back pain | 455 | 276 | 60.7 | 1.04 | 1.29 | 0.63 |  |
|  | q14d | Jaw pain | 454 | 113 | 24.9 | 0.38 | 0.94 | 0.09 |  |
|  | q14e | Headaches | 455 | 172 | 37.8 | 0.43 | 0.93 | 0.16 |  |
|  | q14f | Stomach pain | 453 | 127 | 28 | 0.33 | 0.85 | 0.09 |  |
|  | q14g | Muscle pain with activity | 455 | 221 | 48.6 | 0.88 | 1.25 | 0.43 |  |
|  | q14h | Muscle spasms | 455 | 319 | 70.1 | 1.1 | 1.34 | 0.77 |  |
|  | q14i | Limited activity due to pain | 454 | 209 | 46 | 0.83 | 1.28 | 0.38 |  |
|  | q15a | Bladder or bowel dysfunction | 453 | 232 | 51.2 | 1.06 | 1.36 | 0.54 |  |
|  | q15b | Constipation | 455 | 249 | 54.7 | 0.89 | 1.24 | 0.48 |  |
|  | q15c | Diarrhea | 453 | 141 | 31.1 | 0.44 | 0.97 | 0.14 |  |
|  | q15d | Needing assistance to go to the bathroom | 455 | 194 | 42.6 | 1.24 | 1.68 | 0.53 |  |
|  | q15d | Needing assistance to go to the bathroom | 455 | 194 | 42.6 | 1.24 | 1.68 | 0.53 |  |
|  | q15e | Trouble with bladder control | 454 | 196 | 43.2 | 0.8 | 1.3 | 0.34 |  |
|  | q15f | Increased urination | 453 | 197 | 43.5 | 0.73 | 1.2 | 0.32 |  |
|  | q15g | Loss of appetite | 453 | 204 | 45 | 0.63 | 1.1 | 0.28 |  |
|  | q15h | Nausea | 454 | 68 | 15 | 0.2 | 0.7 | 0.03 |  |
|  | q15i | Burping | 453 | 125 | 27.6 | 0.24 | 0.73 | 0.07 |  |
|  | q15j | Heartburn | 452 | 136 | 30.1 | 0.34 | 0.83 | 0.1 |  |
|  | q15k | Trouble with bowel control | 455 | 129 | 28.4 | 0.54 | 1.12 | 0.15 |  |
|  | q16a | Shortness of breath | 453 | 271 | 59.8 | 1.11 | 1.32 | 0.66 |  |
|  | q16b | Shortness of breath with activity | 451 | 318 | 70.5 | 1.36 | 1.37 | 0.96 |  |
|  | q16c | Breathlessness while speaking | 446 | 250 | 56.1 | 1.12 | 1.38 | 0.63 |  |
|  | q16d | Coughing | 453 | 280 | 61.8 | 1.06 | 1.29 | 0.66 |  |
|  | q16e | Sudden episodes of being unable to speak or breathe | 451 | 135 | 29.9 | 0.57 | 1.13 | 0.17 |  |
|  | q17a | Trouble swallowing | 454 | 259 | 57 | 1.18 | 1.43 | 0.67 |  |
|  | q17b | Difficulty eating food | 454 | 252 | 55.5 | 1.19 | 1.48 | 0.66 |  |
|  | q17c | Excessive saliva | 453 | 259 | 57.2 | 1.09 | 1.41 | 0.63 |  |
|  | q17d | Difficulty chewing | 454 | 213 | 46.9 | 0.96 | 1.41 | 0.45 |  |
|  | q17e | Choking | 454 | 253 | 55.7 | 1.09 | 1.39 | 0.61 |  |
|  | q17f | Needing more time to eat food | 451 | 327 | 72.5 | 1.4 | 1.46 | 1.02 |  |
|  | q17g | Spilling on oneself while drinking or swallowing | 452 | 262 | 58 | 1.01 | 1.38 | 0.58 |  |
|  | q17h | Fear of choking | 453 | 237 | 52.3 | 1.03 | 1.42 | 0.54 |  |
|  | q17i | Trouble swallowing liquids | 454 | 221 | 48.7 | 1.05 | 1.45 | 0.51 |  |
|  | q17j | Difficulty coughing up thick secretions | 453 | 218 | 48.1 | 1.04 | 1.42 | 0.5 |  |
|  | q17k | Drooling | 454 | 216 | 47.6 | 0.83 | 1.31 | 0.4 |  |
|  | q18a | Difficulty speaking | 452 | 267 | 59.1 | 1.52 | 1.61 | 0.9 |  |
|  | q18b | Difficulty communicating | 451 | 246 | 54.5 | 1.32 | 1.51 | 0.72 |  |
|  | q18c | Difficulty using a phone | 448 | 277 | 61.8 | 1.53 | 1.6 | 0.94 |  |
|  | q18d | Slurred speech | 448 | 232 | 51.8 | 1.3 | 1.57 | 0.67 |  |
|  | q18e | Having to repeat oneself when speaking | 448 | 248 | 55.4 | 1.3 | 1.54 | 0.72 |  |
|  | q18f | Fatigue with talking | 447 | 265 | 59.3 | 1.3 | 1.5 | 0.77 |  |
|  |  |  |  |  |  |  |  |  |  |
|  | ***Supplemental Table 1:*** Prevalence, average life impact, and population impact score by symptomatic question | | | | | | | |  |
|  |  |  |  |  |  |  |  |  |  |
